# Restoration of physiologic hemodynamics in the ascending aorta following aortic valve Rreplacement: a 4D flow MR study

**DOI:** 10.1186/1532-429X-18-S1-P346

**Published:** 2016-01-27

**Authors:** Eric J Keller, S C Malaisrie, Jane Kruse, Pim van Ooij, Edouard Semaan, Patrick McCarthy, James C Carr, Michael Markl, Alex J Barker, Jeremy D Collins

**Affiliations:** 1 grid.465264.7Radiology, Northwestern University, Chicago, IL USA; 2 grid.465264.7Division of Surgery-Cardiac Surgery, Northwestern University, Chicago, IL USA; 3 grid.16753.360000000122993507Biomedical Engineering, Northwestern University, Evanston, IL USA

## Background

Previous work has illustrated that blood flow patterns following aortic valve and/or aortic root replacement (AVR/ARR) are dependent on the valve and procedure type. Compared to healthy volunteers, ARR with bioprosthetic and mechanical valves demonstrated increased blood velocities and helicity/vorticity in the ascending aorta, with none restoring physiologic hemodynamics. On-X mechanical valves have unique design features, such as flared inlets and larger length-to-diameter ratios more analogous to the natural left ventricular outflow tract. Thus, we sought to examine the impact of this alternative design on ascending aortic hemodynamics.

## Methods

11 patients (9 men, 45 ± 12 years) with aortic valve disease underwent contrast-enhanced 4D flow MR at 1.5T before and after AVR ± ARR with an On-X mechanical valve prosthesis. 11 age/gender-matched controls (9 men, 42 ± 12 years) were also analyzed to characterize baseline, physiologic flow patterns. 4D flow MR data were corrected for eddy currents, Maxwell terms, and velocity aliasing in Matlab. Peak transvalvular pressure gradients were computed from peak velocity, using the Simplified Bernoulli equation (ΔP = 4v^2^), and aortic hemodynamics were visualized with streamlines (Ensight) and graded separately for helicity by two blinded reviewers (1-3; <180°, 180-360°, >360°). Patients' post-operative pressure gradients and helicity grades were then compared to pre-operative and control values via one-tailed t-tests and Wilcoxon signed ranks tests, respectively.

## Results

**Figure 1:**
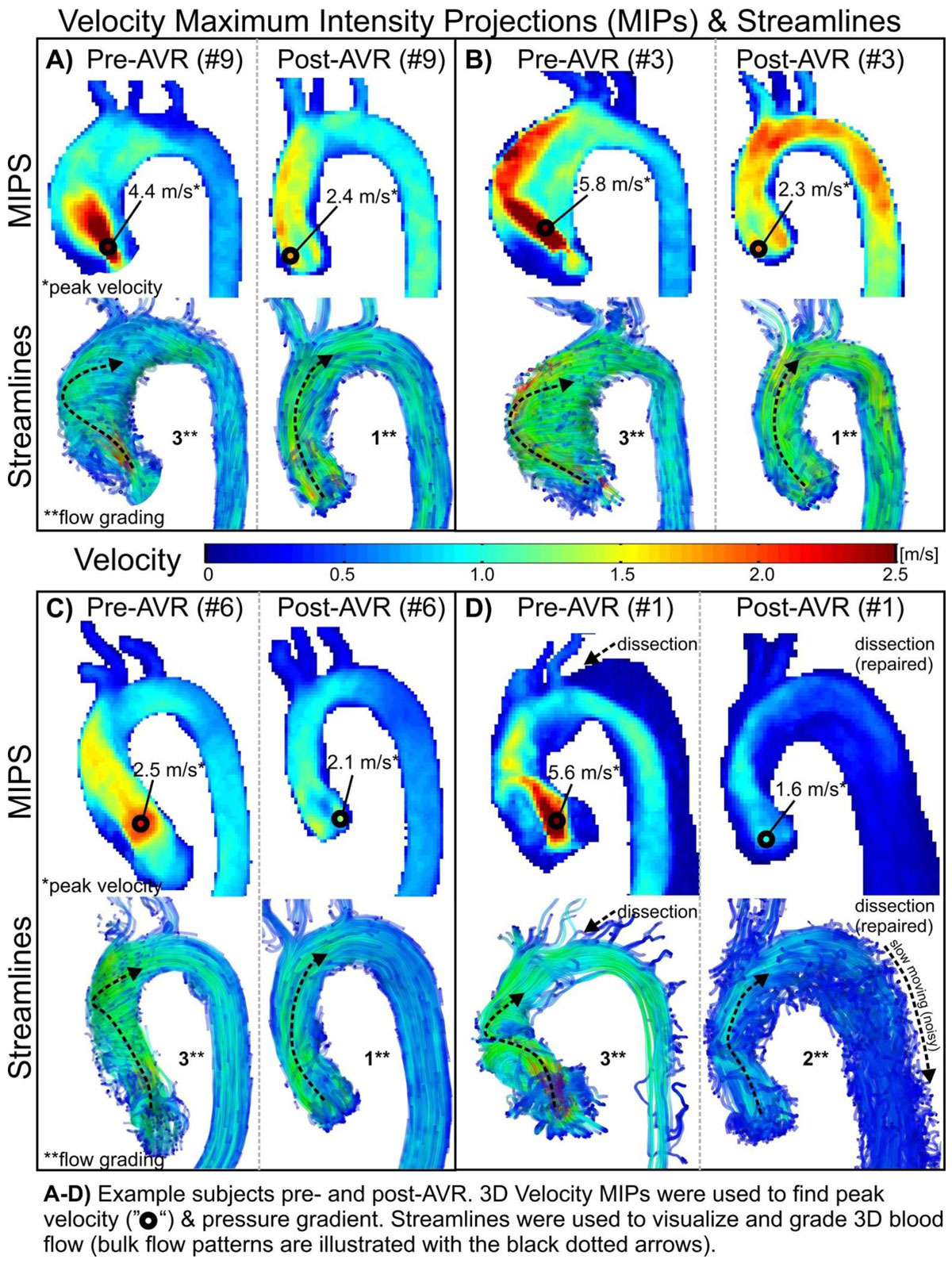
Figure 1

Inter-observer ratings showed good agreement (κ = .77, p < .001). Eccentric, helical flow was identified in all patients pre-operatively, which was significantly reduced following AVR ± ARR [mean grading, 2.7 ± 0.7 (pre) v. 1.5 ± 0.7 (post), p < .05], restoring similar flow patterns to controls (1.1 ± 0.3, p > .05). Although not statistically significant, post-operative flow appeared supra-physiologic with absence of commonly-observed, right-handed, helical flow during systole in some patients (Figure 1). Peak transvalvular pressure gradients (ΔP) were also significantly reduced following AVR ± ARR [54 ± 48 mmHg (pre) v. 18 ± 9 mmHg (post), p < .05], but remained significantly higher than controls (6 ± 1 mmHg, p < .05). See Table 1.

**Table 1:** Transvalvular pressure gradients and helical flow ratings for 11 patients who underwent aortic valve replacement with the MCRI On-X mechanical valve and healthy age and gender matched controls.

Patients				Pre AVR		Post AVR		Controls			
Age	Sex	Intervention	Valve Size (mm)	ΔP (mmHg)	Grading	ΔP (mmHg)	Grading	Age	Sex	ΔP (mmHg)	Grading
58	M	AVR+ARR	27	127	3	10	2	59	M	6	2
29	M	AVR+ARR	27	11	3	23	1	27	M	7	1
43	M	AVR+ARR	25	135	3	21	1	44	M	6	1
29	M	AVR+ARR	27	-	-	7	1	25	M	5	1
55	M	AVR+ARR	25	6	1	6	1	52	M	4	1
48	M	AVR+ARR	27	24	3	9	1	48	M	6	1
44	F	AVR	23	-	-	21	1	37	F	8	1
29	M	AVR+ARR	27	15	3	29	2	29	M	6	1
40	M	AVR+ARR	23	96	3	22	1	40	M	5	1
53	F	AVR	25	42	3	17	2	52	F	8	1
62	M	AVR+ARR	23	34	2	36	3	53	M	9	1
45 ± 12			25 ± 2	54 ± 48	2.7 ± 0.7	18 ± 9	1.5 ± 0.7	42 ± 12		6 ± 1	1.1 ± 0.3

## Conclusions

Preliminary evidence suggests the On-X mechanical aortic valve design may restore physiologic hemodynamics in the ascending aorta in contrast to flow-patterns described in previous reports for other commercially available valve designs. The clinical implications of restoring near physiologic flow patterns after aortic valve surgery are unknown; however, less asymmetric distribution of wall shear stress may slow endothelial cell dysfunction and wall degeneration in the native aorta, reducing risk of future atherosclerotic change and aneurysm formation.

